# Constitutional Factors and Irradiation as Risk Factors for Thymoma: A European Case–Control Study

**DOI:** 10.3390/ijerph21091241

**Published:** 2024-09-20

**Authors:** Linda Kaerlev, Mikael Eriksson, Pascal Guénel, Franco Merletti, Maria Morales-Suárez-Varela, Wolfgang Ahrens, Karl-Heinz Jöckel, Agustin Llopis-Gonzalez, Gun Wingren, Lorenzo Simonato

**Affiliations:** 1 Research Unit of Clinical Epidemiology, Institute of Clinical Research, University of Southern Denmark, 5230 Odense, Denmark; l.kaerlev@dadlnet.dk; 2 Center for Clinical Epidemiology, Odense University Hospital, 5230 Odense, Denmark; 3 Health Promotion, Department of Public Health, Faculty of Health Sciences, University of Southern Denmark, 6705 Esbjerg, Denmark; 4 Department of Oncology, Skåne University Hospital, Lund University, 222 42 Lund, Sweden; mikael.eriksson@med.lu.se; 5 Inserm, CESP (Center for Research in Epidemiology and Population Health), Team Exposome and Heredity, University Paris-Saclay, Gustave-Roussy, 94805 Villejuif, France; pascal.guenel@inserm.fr; 6 Department of Medical Sciences, University of Turin, 10124 Turin, Italy; franco.merletti@unito.it; 7 Research Group in Social and Nutritional Epidemiology, Pharmacoepidemiology and Public Health, Department of Preventive Medicine and Public Health, Food Sciences, Toxicology and Forensic Medicine, Faculty of Pharmacy and Food Sciences, Universitat de València, Av. Vicent Andrés Estelles s/n, 46100 Burjassot, Spain; agustin.llopis@uv.es; 8 CIBER of Epidemiology and Public Health (CIBERESP), Carlos III Health Institute, Av. Monforte de Lemos 3–5 Pabellón 11 Planta 0, 28029 Madrid, Spain; 9 Department of Epidemiological Methods and Etiologic Research, Leibniz Institute for Prevention Research and Epidemiology—BIPS, 28359 Bremen, Germany; ahrens@leibniz-bips.de; 10 Institute for Medical Informatics, Biometry and Epidemiology, University Clinic Essen, 45147 Essen, Germany; k-h.joeckel@uk-essen.de; 11 Division of Occupational and Environmental Medicine, Department of Clinical and Experimental Medicine, Linköping University, 581 83 Linköping, Sweden; gun.wingren@gmail.com; 12 Department of Cardiovascular, Thoracic Sciences and Public Health, University of Padova, 35122 Padova, Italy; lorenzo.simonato@unipd.it

**Keywords:** thymoma, cancer, radioactivity, X-ray, radiation-induced, constitution, comorbidity, risk factors, case–control study

## Abstract

Little is known about the aetiology of thymoma. This study aims to identify medical risk factors for thymoma as a systematic approach to new hypotheses on the aetiology of this disease. A European multi-centre case–control study was conducted from 1995 to 1997, including incident cases aged 35–69 years with thymoma. Altogether, we accepted 85 cases and 3350 controls, of which we interviewed 77 cases and 2071 population controls about constitutional factors, medical examinations, and former diseases. Odds ratios (ORs) with 95% confidence intervals (CI) were calculated. Medical examinations with X-ray or radiotherapy performed >20 times at least one year before the thymoma diagnosis indicated a possible risk factor for thymoma (OR 1.58, 95% CI 0.93–2.69). Having the first radiotherapy treatment at least one year before the thymoma diagnosis yielded an OR for thymoma of 2.39; 95% CI (0.96–5.99), and if it was at least five years before, the OR for thymoma was 2.81; 95% CI (1.03–7.72). Having a red/auburn hair colour was associated with thymoma, (OR 3.6, 95% CI 1.4–9.5) whereas having pigmented skin was slightly associated with thymoma (OR 1.8, 95% CI 0.8–3.8). Over twenty instances of X-ray examinations or radiotherapy were identified as potential risk factors for thymoma, along with certain constitutional factors. The observed correlations between benign tumours and thymoma could stem from an inherent predisposition to tumour development or result from detection bias. Given that this is the initial analytical study examining medical risk factors for thymoma, all of the results should be approached with caution, acknowledging the possibility that some findings might be incidental.

## 1. Introduction

Thymomas are rare tumours originating from thymic epithelial cells. The majority retain a benign character, but, potentially, they may behave as malignant tumours with a significant risk of recurrence, and some of them develop into well-differentiated carcinomas. In Europe and the USA, the incidence of thymoma is around 0.1–0.3 per 100,000 in males and females, and the incidence rate increases with age [1,2,3]. In Europe, the frequency of thymoma has measured by the RARECARE and RARECAREnet projects, as described in a paper by Siesling in 2012 [4]. In these data, the oldest age group (65 years or older) has an incidence rate of 0.42 per 100,000 compared to an incidence rate of 0.19 per 100,000 in the 25–64-year age group, as reported in a review from 2016 by Scorsetti et al. [1]. Thymomas may precede autoimmune diseases or B-cell non-Hodgkin’s lymphoma, and subsequent soft tissue sarcoma has also been reported in a US study, apparently as a consequence and not the cause of the thymoma [5]. The US study also suggested that there may be genetic risk factors due to a higher incidence in Asian, Pacific Islander and black populations than in white populations [5].

In a study, 47 out of 85 thymoma cases had myasthenia gravis (n = 33), Hashimoto’s thyroiditis (n = 4), Isaac’s syndrome (n = 3), Morvan’s syndrome (n = 2), pure red cell aplasia (n = 2), systemic lupus (n = 2), lichen planus (n = 2), and other rare types (one case each) [6]. The aetiology of thymoma remains largely unknown. An Epstein–Barr Virus (EBV) infection has been suspected of being a risk factor for thymic carcinomas, and the EBV genome was present in Chinese thymoma patients [3]. A Japanese case report suggested a relationship between an HTLV-1 carrier state and thymoma [7], and an Italian case report suggested a relationship between HIV and thymoma due to a case of thymoma among 49 different tumours in a group of patients with HIV infection [8]. In a UK birth cohort register study, which did not distinguish between thymomas and thymic carcinomas, persons born during the Second World War were found to be at low risk [9]. Some studies suggest that irradiation exposure such as X-ray, CT-scan examinations, and radiation therapy is a risk factor, and there are reports of synchronous thyroid and thymic malignancy following childhood irradiation [10,11,12,13,14]. Very few studies investigated chemical exposures, but an animal study shows that vinyl carbamate may induce thymoma when administered in the first weeks after birth [15]. A thymoma case in a worker exposed to fluorocarbons in an automobile plant has been reported [16]. A former publication from our study on thymomas, as well as a case series from France, suggested tobacco as a risk factor [17,18]. We performed the first large European multicentre case–control study on thymoma, focusing on medical conditions and X-ray exposure in relation to thymoma.

## 2. Material and Methods

This study was conducted in accordance with the principles of the Declaration of Helsinki. Participation was voluntary, and all patients provided written informed consent prior to enrolment. This study was approved by ethical committees or boards in all the participating countries/areas. This study has been described previously in detail [19]; a brief summary is included here.

A multi-centre case–control study was conducted in 10 European countries. During a two-year period between 1995 and 1997, incident cases of thymoma were collected. We evaluated the possible medical and environmental risk factors of rare cancers located at seven different sites [small bowel, thymus, bone, gall bladder, male breast, skin (mycosis fungoides), and eye (malignant melanoma)] using the same data collection instruments. Incident cases of thymoma together with population controls, who were 35–69 years old when they were diagnosed, were recruited in different regions in five of the countries: Denmark (the whole country), Sweden (Umeå, Örebro/Uppsala, Linköping, Lund), France (Calvados, Côte d’Or, Doubs, Hérault, Isère, Manche, Bas-Rhin, Haut-Rhin, Somme, Tarn), Germany (Hamburg, Bremen, Essen, Saarland), and Italy (Turin, Florence, Eastern Veneto). The combined populations served by the participating hospitals of each of these areas were, approximately, 5 million (Denmark), 4 million (Sweden), 6 million (France), 4 million (Germany), and 3 million (Italy), during the incident case collection time-period between 1995 and 1997.

All histologically and clinically verified incident epithelial thymic tumour cases with topography code C37.9 according to ICD-O-2 1990 [20,21] and with morphology code 8580/0–8580/3 were identified based on repeated requests (with a recommended interval on every three months or less) to hospital and pathology departments and by similar frequent checks of regional or national cancer and pathology registers, and these were enrolled in this study. A secondary review of the histological slides was conducted by a reference pathologist (PJ), who was blinded to exposure status. Only cases of histologically confirmed thymoma or thymic carcinoma, as validated through this review process, were included in this study.

This study was population-based in these five countries and sub-regions. The study population has been described in detail in our method papers (overall method in [19] and control selection further described in [22]). A short description adapted from these papers follows. Population controls were randomly selected from population registries at each centre and frequency matched with cases in 5-year age bands by sex and centre to obtain a balanced data set. The controls were selected at intervals throughout the 2-year study period, e.g., every three to six months to achieve incidence density sampling, and they were selected from population registers (Denmark, Italy, and Sweden), from electoral rolls (France), and from municipality registers (Germany). As control extraction was relatively expensive with this method in Germany, a large pool of potential controls was selected at the beginning of the study period, and the controls were subsequently selected from this pool as published previously [19]. The goal was to achieve at least four controls per case for the most frequent of the seven rare cancer types within each stratum. Since the control groups for all seven cancer types were pooled for the analyses of each cancer type to obtain the best precision score, a control–case ratio higher than four was obtained in the thymoma study.

One hundred and twenty incident thymoma cases were recruited; however, five were judged as not eligible by the reviewing pathologist and were excluded from analysis. Of the 115 remaining cases, 93 were classified as “definitive”, and 22 were classified as “possible”. Of the 93 cases, 85 patients had a locally made histological diagnosis of thymoma. Of these 85 patients, all necessary data were available for 77, which were finally included in this study; these are what we called definitive epithelial thymic tumour patients. Of the 3350 controls that accepted participation in this study, 2071 responded to the interview, and complete data were available.

As soon as the cases and controls were enrolled, trained interviewers conducted a face-to-face or telephone interview. If the index person was dead or too ill to participate, next-of-kin were interviewed if possible [23].

### 2.1. Interview

The interview followed a standardised questionnaire specifically developed for this study. Basic information was obtained about demographic characteristics, tobacco smoking, the average daily intake of specific types of alcoholic beverages, educational qualifications, adult height and weight, and reproductive conditions. Information was also collected about medical treatment with radiotherapy and radioactive substances and use of corticosteroid hormones for more than six months.

Questions were asked about previous mumps, herpes, typhus, asthma, dermatitis, urticaria, diabetes, psoriasis, thyroid disease, bone disease, inflammatory bowel disease, gallstones, hepatitis, or jaundice, and the disease categories (ICD-8 code) were set as follows: other benign neoplasms (21–22) at least one year before the thymoma diagnosis for cases and at least one year before the interview for controls, brain and nerve system diseases (32–35), ear disease (38), upper gastrointestinal ulcer/functional disorder (53), breast/ovarian/parametrium disorder (61), cardiac disease (41–42), respiratory tract infection (46–49), infection (0–13), urinary tract diseases (58–59), and osteoporosis (72). We also asked about the age at onset/treatment and whether the diagnosis was made by a physician. Only diseases reported as confirmed by a medical doctor and with at least one year of latency time before the incident thymoma diagnosis were included in the analyses.

We screened for the subject having a total number of more than 20 diagnostic X-ray examinations (median of exposure frequency for the sample), and if so, we sought information about the total number the subject had ever had regardless of condition, but no dental examinations or non-medical examinations, e.g., in shoe shops, were included. No separation between ultrasound and X-ray was indicated in the questionnaire. CT scan is a kind of X-ray, but it was not possible to make a distinction between CT scans and other X-rays. The type of X-ray investigation and organ target were not specified in the questionnaire.

For each medical condition with X-ray examination mentioned, we asked for the number of examinations in total for this condition and, if affirmative, the age at the first examination (question from the questionnaire in the Appendix A). In the analyses, we only focused on the association between thymoma and exposure to diseases or to medical X-ray examinations performed with at least a one-year latency time before the incident diagnosis of thymoma or the reference date (date of interview) for the controls and for X-ray, in another analysis, with at least a five-year latency time to diminish recall bias and reverse causality and detection bias. Participants who had undergone fewer than 20 diagnostic X-ray examinations were not queried further regarding this matter and were classified as unexposed.

Regarding gallstone disease, we also asked whether an ultrasound or X-ray was performed, whether there was surgery performed, and, if so, age at the first surgery.

### 2.2. Statistical Methods

We calculated age at diagnosis and time to interview for cases and we calculated age at interview for population controls. We further calculated demographic characteristics of the cases and controls. Adjusted odds ratios (ORs) with 95% confidence intervals (CIs) were obtained by unconditional logistic regression. All analyses were adjusted for matching variables of gender, country (five categories), and year of birth (as a continuous variable) [24,25]. A frequency matching sampling design was used to assure that cases and controls had the same distributions over strata defined by matching factors, and, therefore, a matched-pair conditional logistic regression analysis was not needed. However, frequency matching resulted in the controls being more similar to the cases than they were in the source population, biasing the OR towards 1, and we therefore needed to control for the matching variables of age, gender, and region of living in the unconditional logistic regression analyses [26]. No established risk factors for thymoma are known in the literature as a relevant confounder besides these matching variables.

We investigated the relationship between thymoma and exposure to diseases or medical X-ray examinations. Our analyses considered exposures occurring at least one year before the diagnosis of thymoma or the reference date for controls. For X-ray exposures, we also evaluated those occurring at least five years before. For X-ray, we further adjusted for having had benign neoplasms at least one year before the thymoma diagnosis among cases and at least one year before the interview among the frequency-matched population controls. In sensitivity analyses, we reran all the analyses with the exclusion of Sweden, the country with the lowest case response rate.

## 3. Results

In all, we identified 85 patients with a locally made histological diagnosis of thymoma who were evaluated as eligible by the reference pathologist; these are presented in Table 1. A total of 74 cases were classified as cases with “definite” epithelial thymic tumours. A further 11 patients were classified as “possible” thymoma cases either according to the referent pathologist or because no histological sections were available for review. The collected and reviewed HE-stained sections did not allow the pathologist to discriminate between benign and malignant tumours. Thus, 85 cases of thymoma and 3350 population controls were accepted for this study, and 8 (9%) of these were not interviewed (6 refused to participate, 1 could not be contacted, and 1 case was dead without a next-of-kin to be interviewed), leaving 77 cases with interviews (91% response; 43 male; 34 female; index person interview, 75; next-of-kin interview, 2).

In the control group, 2071 responded (62%; 1447 males; 624 females; index person interview, 2047; next-of-kin interview, 24). The response rate for controls ranged from 55% in Denmark and Germany to 77% in France. We found only small differences in age and sex between responding and non-responding controls.

Table 2 presents the demographic profiles of both cases and controls. It shows that there were no significant differences in age distribution at the time of the interview, nor in body mass index (BMI) parameters such as minimum or maximum BMI, BMI at age 35, and BMI from 1 to 5 years prior to the interview, between the cases and controls. The average duration from diagnosis to interview for cases was 0.69 years (ranging from a minimum of 0 to a maximum of 2.55 years). The average time from diagnosis to interview among cases, broken down by country (in years), was as follows: Denmark—0.52, Sweden—0.40, France—0.60, Germany—0.46, and Italy—0.99. Notably, a higher proportion of males and individuals with lower educational levels was observed among the cases compared to the controls. In all our analyses, including sub-analyses, adjustments were made for country, sex, age (year of birth), and educational level.

Constitutional factors such as time to maximum height and eye colour were not associated with thymoma (Table 3). Having a red/auburn hair colour, (OR 3.6, 95% CI 1.4–9.5) was associated with thymoma. Having pigmented skin was slightly associated with thymoma (OR 1.8, 95% CI 0.8–3.8). The OR for hair colour, OR 2.01; 95% CI (0.44–9.27), and for skin colour, OR = 2.88; 95% CI (0.34–24.36), were still increased twofold after exclusion of the countries with the highest ORs (Italy for hair colour, and France for skin colour) but not significantly increased due to lack of power in this study.

The risk of thymoma following previous diseases is described in Table 4. Thymoma was statistically significantly associated with benign neoplasms diagnosed at least one year before the thymoma diagnosis (five myoma uteri, one intestinal polyp, one ovarian cyst, one breast fibroma, one tendon cyst, one cyst of the neck, and one urinary bladder papilloma reported among seven cases), as well as with ear diseases. No association with other disorders was observed. A statistically non-significant association between thymoma and prior upper gastrointestinal tract diseases (ulcers or functional disorders of the oesophagus/stomach/duodenum) was seen.

We could not demonstrate any association with infertility or testis surgery among men nor use of oral contraceptives or hormonal replacement therapy, experience of miscarriage, or terminated pregnancy among women.

The OR for thymoma among subjects who had >20 X-ray examinations at least one year before the thymoma diagnosis was 1.58 (95% CI: 0.93–2.69). For those who had their first radiotherapy at least one year before their thymoma diagnosis, the OR was 2.39 (95% CI: 0.96–5.99); this OR was raised to 2.81 (95% CI: 1.03–7.72) when taking into account a minimum of a five-year latency time before the thymoma diagnosis (Table 5). When we further adjusted for having had benign neoplasms at least one year before the thymoma diagnosis among cases and at least one year before the interview among population controls, the association was only borderline statistically significantly increased.

The injection of radioactive substances with a restriction to ≥one or ≥five years before the thymoma diagnoses showed an OR of 0.79 and 1.11, respectively, both based on nine cases, but had wide confidence intervals and did not reach statistical significance.

## 4. Discussion

To the best of our understanding, this research represents an inaugural large-scale, multicentre, population-based case–control study dedicated to investigating potential medical and environmental risk factors associated with thymoma. This study is characterised by a scarcity of existing etiological hypotheses. Employing a standardised methodology, we successfully identified thymoma (along with six other rare cancer types) and gathered data on various exposures across numerous European countries. This study has made significant contributions, particularly for other cancer types within the European research framework. For instance, it has played a crucial role in generating evidence that has led to the classification of welding and 1,2-dichloropropane as human carcinogens, as recently documented in a published epidemiological paper [27]. For thymoma, we previously analysed tobacco and alcohol, and we suggested tobacco as a risk factor [18].

In our analysis, we discovered a correlation between receiving radiotherapy or undergoing more than 20 X-ray examinations at least a year prior and a subsequent diagnosis of thymoma. This association persisted even when considering a latency period of five years before the thymoma diagnosis, suggesting that irradiation could be a potential risk factor for thymoma. However, we did not find a similar risk associated with the injection of radioactive substances, and it may be that the explanation of the radiation–thymoma finding may be “confounding by indication”, i.e., that the underlying disease for which X-ray was used could have been a risk factor for thymoma and not the irradiation itself.

The risk of thymoma was associated with red/auburn hair colour, especially in Italy. The risk of thymoma was also associated with pigmented skin, especially in France. Although these findings are limited to these countries in our data, they suggest that genetic components may play a role in the occurrence of thymoma. A genetic component in thymoma risk was also suggested by two cancer registry studies showing that the incidence of thymoma was highest among Asian/Pacific Islander and non-Hispanic black individuals and thus supports the finding [5,28].

An association with previous benign neoplasms other than thymoma may simply suggest a tendency to develop benign tumours and not necessarily point to a genetic component, e.g., PTEN mutation, as the aetiological factor. Associations between thymoma and prior upper gastrointestinal tract diseases (ulcers or functional disorders of the oesophagus/stomach/duodenum) were seen, also with previous ear diseases, which may be random findings.

### 4.1. Methodological Considerations and Potential Biases

Thymic tumours are a heterogeneous group of malignancies with varied clinical presentations, and most studies of thymoma have been based on small numbers of clinical cases recruited at single centres. The most common types are thymoma and thymic carcinoma, but, overall, they remain rare cancers with no clear aetiology. This presents a significant challenge, as both the low incidence and the lack of prior information on potential risk factors complicate efforts to address the underlying causes of this rare cancer.

The ideal method for investigating potential causal agents in the development of a condition is a case–control study, as it allows for a direct comparison of environmental exposures and the development of thymoma. However, due to the rarity of the condition, implementing such studies proves difficult.

Analyses of the disease primarily depended on participants’ ability to recall past information, which may have been less accurate among healthy controls compared to tumour patients. To minimise potential bias, we only included data on previous medical conditions confirmed by a physician (clinical history: X-ray examination and chest examination). Two papers report the long-term effects of radiation in infants who received thymic radiation for benign enlarged thymus glands. None of the infants developed thymoma, although there was an increased rate of subsequent thyroid carcinoma [29] and breast cancer [30]. In addition, there is no reported increase in the rate of thymoma following radiotherapy to the chest for lung or breast cancer or Hodgkins Lymphoma [5].

Since ultrasound does not emit any ionising radiation, exposure to this investigation may have served as an indicator for recall bias in our study. We were, however, solely able to calculate the odds ratio for ultrasound examinations as a part of gallstone examinations (ultrasound or X-ray examination), and thus we could not distinguish between these two examination methods. However, this inability to separate the two probably means that any ORs for radiation through “X-ray/ultrasound” would have been diluted and did not falsely strengthen any of the risks presented. The possible effect would be of an underestimation of the risk.

One of the main weaknesses of this study is that no concrete radiation exposure dose is available. In this study, only conventional X-ray examinations and X-ray CT scans were considered for both cases and controls, and the radiation dose used was not taken into account, which introduces an information bias. When studying radiation exposure, not only are exposure times important but also the dose per exposure. For example, even in 20 conventional X-ray exposures, the total dose (1 mSv level) would be lower than the dose of one conventional CT examination. Dose calculation is, however, almost impossible when relying upon subjects’ memory and knowledge, since radiation doses vary so much between different examinations, and even CT scans may be performed with a high or low radiation dose. Also, as previously mentioned, the type of X-ray investigation and organ target were not specified in the questionnaire, but the most common investigation, chest X-ray, may affect the thymus. We believe that it is likely that the mixture of exposures may be rather similar between cases and controls.

Detection bias might arise from scans for other conditions, such as benign neoplasms that we have identified in this study and the possibly of secondary malignancies [31] from the Swedish Cancer registry to investigate this possible link, reporting a twofold increased risk of second malignancy in patients with thymoma compared to the general population. The literature does not clearly describe how often thymoma is found incidentally [32]. Thymoma symptoms—such as chest pain or pressure, coughing, and shortness of breath—are common and non-specific. This non-specific presentation may influence the heterogeneity of the reported cases, even though all the cases in this study are newly diagnosed.

We mitigated the risk of reverse causation by coding irradiation exposure in the five years before diagnosis or the reference date as unexposed. The effects of radiation exposure are typically long-term; however, in our case, the reliable information we had access to only went back five years. Therefore, we had to limit our analysis to this period.

Controlling for confounding factors is challenging because the aetiology of thymoma is largely unknown. A low educational level was associated with thymoma, and social factors could affect disease-reporting accuracy and access to medical services, potentially confounding our results. However, adjusting for education in a sub-analysis did not alter our estimates. Another possible confounding factor that we did not consider was solid organ transplant [33], which could be interesting to assess in the future.

Information bias might have arisen from interviews with proxy respondents when the case participant was too ill to participate. This occurred more often for thymoma cases (2.6%) than for controls (1.2%), which could have led to misclassification of medical conditions. Excluding the few next-of-kin from sub-analyses did not affect the results.

Histological review by an experienced pathologist was a key strength of our study, ensuring the inclusion of histologically confirmed thymoma cases and reducing diagnostic variability across countries.

Despite the European multi-centre design, the small case group, due to the disease’s rarity, limited statistical power, as indicated by wide confidence intervals. Consequently, some associations might have been undetected.

Delayed data submission by some national registries might have postponed thymoma case ascertainment, potentially increasing cancer stage and decreasing performance status at recruitment, which could partly explain variations in response rates across countries. Sensitivity analyses excluding Sweden, with its low response rate for thymoma cases, did not significantly alter estimates but did reduce this study’s power.

In a methodology paper, we compared colon cancer controls with population controls in our rare cancer studies, including thymoma. Colon cancer patients, frequency-matched by gender and 5-year age groups, had a similar disease profile to population controls. The control groups in Spain and Portugal were hospital-based, which might have underestimated thymoma risk. We thus used data from five countries with population controls and excluded Spain and Portugal. Minor differences in age and sex between responding and non-responding controls suggest that selection bias based on these factors was minimal.

For this European study, we pooled data from five standardised parallel case–control studies on seven rare cancers collected simultaneously in Europe using uniform data collection schedules and procedures during the same time period. A 2016 review by Scorsetti et al. [1] noted a higher incidence of thymoma in Southern Europe compared to Northern Europe, consistent with our findings. Our study only included thymoma cases aged 35–69, but incidence increases with age, peaking in those aged 65 and older. We present only the number of thymoma cases and the total population in participating countries and regions, not incidence rates.

Controls included slightly more males (70%) than thymoma cases (56%). All analyses were adjusted for gender, year of birth, and country. As there are no established risk factors for thymoma, controlling for other potential confounders is difficult. Previous research has suggested tobacco smoking and heavy alcohol consumption might be risk factors.

### 4.2. Ionising Radiations

Regarding ionising radiation, 27% of the controls had over 20 X-rays during their lifetime, excluding dental X-rays. This high percentage likely reflects routine or screening examinations rather than over-reporting. Despite high reported X-ray numbers among both thymoma cases and population controls, we found no evidence of over-reporting.

Due to incomplete regional and age group participation in the five countries, we could not compare this percentage with other studies. Excluding exposures within five years of the interview, the association between medical radiation and thymoma remained, suggesting a possible real association or a common pathogenesis involving conditions leading to X-rays, such as genetic syndromes linked to benign tumours. Further adjustment for benign neoplasms did not significantly alter the association due to a broader confidence interval. We did not conduct a dose–response analysis for X-rays due to the small number of exposed cases (n = 22). Although there are reports of synchronous thyroid and thymic malignancies following childhood radiation treatment, these are based on case reports [14,34]. Another explanation could be confounding by indication, where underlying diseases or inflammation requiring X-rays might be the actual risk factor for thymoma rather than the radiation itself.

For other medical conditions, we found associations with diseases previously reported as co-morbidities with thymoma, such as other benign neoplasms, but not with autoimmune disorders. This supports the view that these disorders are likely a consequence of thymoma rather than a cause. Increased medical surveillance for thymoma symptoms might lead to more diagnoses of common or chronic conditions, potentially causing detection bias. We considered cases exposed within one year before diagnosis and controls exposed within one year before the interview as unexposed. An association with ear diseases (OR 6.0) based on three cases was found, but this may be a spurious finding.

The lack of excess risk for some rare medical conditions, such as inflammatory bowel disease or liver cirrhosis, should not be considered definitive due to the low prevalence of these diseases.

### 4.3. Other Medical Conditions

We observed an association with diseases previously reported as co-morbidities of thymoma, such as other benign neoplasms, but did not find a high odds ratio for autoimmune disorders. This supports the prevailing view that autoimmune disorders are likely a consequence of thymoma rather than a cause. Due to increased medical surveillance prompted by early thymoma symptoms, common and chronic conditions, such as ear or oesophageal diseases, may be more frequently diagnosed among thymoma cases than controls. This can introduce detection bias and lead to spurious associations between these conditions and thymoma, particularly when thymoma is diagnosed shortly after other diseases. To address this, we considered cases exposed within one year before the thymoma diagnosis and controls exposed within one year before the interview as unexposed. Nevertheless, an association was observed with ear diseases (odds ratio 6.0) based on three cases, which could potentially be a spurious finding.

## 5. Conclusions

This study is the first population-based analysis specifically examining thymoma. The main findings suggest that having more than 20 X-ray exams or undergoing radiotherapy could be potential risk factors for thymoma. Additionally, there appears to be a link between having red or auburn hair and thymoma, with a slight association observed in those with pigmented skin. The increased risk of ear diseases may be due to detection bias. The connection between thymoma and other benign tumours may reflect a general tendency to develop such tumours, and caution is needed when interpreting these findings, as they could be spurious. Although this study could not differentiate between conventional X-rays and CT scans and had limited statistical power, it suggests a possible association between ionising radiation and an increased risk of thymoma. However, due to the rarity of thymoma, the results may not have immediate preventive implications, though it is worth noting that various epithelial tumours are linked to radioactivity.

## Figures and Tables

**Table 1:** Recruitment of cases with thymoma and controls by country.

Case–Control Status	Denmark, N	Sweden, N	France, N	Germany, N	Italy, N	Total, N
Cases						
Eligible	11	14	25	11	24	85
Dead or too ill to participate	-	-	-	-	1	1
No contact or refused to participate	1	4	-	-	2	7
All cases interviewed	10	10	25	11	21	77
Index case subjects interviewed	10	10	23	11	21	75
Surrogate case subjects interviewed	-	-	2	-	-	2
Telephone interview	9	10	1	0	0	20
Time, diagnosis/interview, mean, years, range	0.52 0.31–0.88	0.40 0.17–0.78	0.61 0.02–2.03	0.46 0.15–1.33	0.99 0.03–2.55	0.65 0.02–2.55
Controls						
Population (P)	P	P	P	P	P	P
Eligible	583	407	630	1325	405	3350
Dead or too ill to participate	1	-	4	46	4	55
No contact or refused to participate	262	177	141	546	98	1224
All controls interviewed, N, %	320, 55%	230, 57%	485, 77%	733, 55%	303, 75%	2071
Index control subjects interviewed	318	229	477	724	299	2047
Surrogate control subjects interviewed	2	1	8	9	4	24
Telephone interview, N, %	312, 98%	227, 99%	55, 11%	50, 7%	40, 13%	684, 26%

**Table 2:** Selected characteristics of 77 cases of thymoma and 2071 controls.

	Cases		Controls	
	N	%	N	%
All subjects	77		2071	
Sex, male	43	55.8	1447	69.9
Age at diagnosis (years)	55.6		-	
Mean age at interview, years	56.3	-	54.5	-
Age at interview (years)				
35–49	22	28.6	721	34.8
50–59	26	33.8	557	26.9
60–69	28	36.4	747	36.1
Surrogate interview	2	2.6	24	1.2
Married	65	84.4	1621	78.3
Educational status, low ^1^	29	37.7	567	27.5
BMI 1–5 years ago, mean ^2^	25.2	-	25.3	-
BMI at age 35	23.7	-	23.7	-
Maximum BMI ^3^	27.0	-	26.9	-
Minimum BMI ^3^	20.9	-	21.4	-

^1^ Left school at the age of 15 years or earlier; no further education. ^2^ BMI 1–5 years prior to diagnosis for cases or to the date of interview for controls. ^3^ Maximum and minimum BMIs ever had.

**Table 3:** Risk of thymoma according to constitutional factors.

Constitutional Factor		Cases, N	Controls, N	OR (95% CI) ^1^
Time reaching maximum height ^2,3^	Same time	55	1347	1.0
	Earlier	12	339	0.8 (0.4–1.7)
	Later	8	261	0.8 (0.4–1.4)
Colour of the eyes ^4^	Blue/grey/green/hazel	46	1380	1.0
	Brown/black/other	31	675	1.1 (0.7–1.9)
Colour of the hair ^5^	Fair/blonde	16	739	1.0
	Red/auburn ^7^	7	61	3.6 (1.4–9.5)
	Brown	36	838	1.4 (0.7–2.6)
	Black	16	336	1.5 (0.7–3.3)
	Other	2	83	0.7 (0.2–3.4)
Colour of the skin ^6^	White	63	1847	1.0
	Non-White ^8^	13	188	1.8 (0.8–3.8)

^1^ Odds ratio with 95% confidence interval adjusted for country, year of birth, and sex. ^2^ Reached maximum height at the same time as the friends of the same age? ^3^ Information missing for two cases and 124 controls. ^4^ Information missing for 16 controls. ^5^ Information missing for 14 controls. ^6^ Information missing for one case and 36 controls. ^7^ Five of the seven red-haired cases were from Italy, and out of the twenty-one Italian cases, five were red-haired. ^8^ Twelve of the thirteen non-white cases were from France, and twelve out of the twenty-five French cases were non-white.

**Table 4:** Risks ^1^ for thymoma regarding certain specified diseases.

Specific Diseases and More General Disease Groups Having Had (≥1 Year Before the Thymoma/Reference Date) Y/N ^2^	Cases, N	Controls, N	Adjusted OR ^1^	95% CI
Mumps	26/25	834/683	0.86	0.49–1.53
Herpes	10/65	164/1859	1.62	0.81–3.25
Typhus	1/76	38/1998	0.47	0.06–3.5
Asthma	4/73	146/1890	0.69	0.25–1.93
Dermatitis	16/57	356/1658	1.24	0.70–2.19
Urticaria	3/70	122/1884	0.54	0.17–1.75
Diabetes ^3^	1/76	99/1945	0.24	0.03–1.77
Psoriasis	3/74	90/1944	0.89	0.27–2.91
Thyroid disease	5/69	145/1874	0.90	0.35–2.35
Bone disease	9/62	198/1799	0.88	0.42–1.85
Inflammatory bowel disease	3/73	56/1966	1.18	0.35–3.91
Gallstone	6/71	146/1885	0.82	0.35–1.97
Gallstone ultrasound or X-ray	5/72	128/1910	0.82	0.32–2.11
Gallstone (operated)	5/72	96/1933	0.99	0.38–2.57
Hepatitis or jaundice	3/71	171/1855	0.42	0.13–1.37
Disease categories				
Other benign neoplasms, ICD-8: 21–22 (other than thymomas) ^4^	7	39	3.88	1.61–9.37
Brain and nerve system, ICD-8: 32–35	2	38	1.49	0.35–6.42
Ear diseases, ICD-8: 38	3	12	6.09	1.64–22.65
Upper gi-tract disease, ICD-8: 53	6	85	2.26	0.92–5.55
Breast/ovarian/parametrii, ICD-8: 61	2	7	3.93	0.75–20.53
Cardiac disease, ICD-8: 41–42	1	95	0.30	0.04–2.19
Resp. tract infections, ICD-8: 46–49	2	73	0.80	0.19–3.39
Infectious diseases, ICD-8:0–13	6	120	1.21	0.51–2.87
Urinary tract diseases, ICD-8: 58–59	4	66	1.67	0.58–4.76
Osteoporosis, ICD-8: 72	2	89	0.83	0.20–3.50

^1^ Odds ratio with 95% confidence interval, adjusted for country, year of birth, and sex. ^2^ Cases with exposure within one year before the thymoma diagnosis and population controls with exposure within one year before the interview were regarded as unexposed. ^3^ Diabetes 1 and diabetes 2 combined due to only one case. ^4^ Seven cases had eleven tumours altogether: five myoma uteri, one intestinal polyp, one ovarian cyst, one breast fibroma, one tendon cyst, one cyst of the neck, and one urinary bladder papilloma.

**Table 5:** Risks ^1^ for thymoma related to exposure to medical radiation.

Medical Condition	Cases N	Controls N	Adj. OR ^1^	95% CI	Adj. OR ^2^	95% CI
X-ray examinations >20 times y/n (first ≥1 years ago) ^3^	22/49	553/1467	1.58	0.93–2.69	1.59	0.93–2.71
X-ray examinations >20 times y/n (first ≥5 years ago) ^4^	20/51	526/1494	1.46	0.85–2.52	1.47	0.85–2.54
Radiotherapy y/n (first ≥1 years ago) ^3^	6/67	67/1972	2.39	0.96–5.99	2.36	0.94–5.93
Radiotherapy y/n (first ≥5 years ago) ^4^	5/68	50/1989	2.81	1.03–7.72	2.67	0.97–7.38
Having had an injection of radioactive substances y/n (first injection ≥1 years ago) ^3^	9/59	290/1693	0.79	0.38–1.63	0.79	0.38–1.63
Having had an injection of radioactive substances y/n (first injection ≥5 years ago) ^4^	9/59	225/1758	1.11	0.53–2.29	1.10	0.53–2.27

^1^ Odds ratio with 95% confidence interval, adjusted for country, year of birth, and sex. ^2^ Adjusted for country, year of birth, and sex and further adjusted for having had other benign neoplasms more than 1 year before the thymoma diagnosis, ICD-8: 21–22. ^3^ Cases with exposure within one year before the thymoma diagnosis and population controls with exposure within one year before the interview were regarded as unexposed. ^4^ Cases with exposure within five years before the thymoma diagnosis and population controls with exposure within five years before the interview were regarded as unexposed.

## Data Availability

Due to the sensitive nature of the data collected, ethical restrictions prohibit us from making the minimal data set publicly available as these are person-related data. Each study centre received approval by the corresponding local ethics committees, and participants did not provide consent for data sharing. Data can only be accessed by registered scientists who are authorised to access the confidential data with an individual account and an individual password. Statistical analyses are performed on the CDS. Data will be made available upon personal request, and all requests need approval by the study’s Steering Committee. Interested researchers can contact the Rare Cancer consortium. A mailed request must be mailed to all three of the following three mail addresses, l.kaerlev@dadlnet.dk; lkaerlev@health.sdu.dk; and roersoe@gmail.com, to request data access for any of the seven rare cancer sites in the rare cancer study (thymoma, male breast, small bowel, biliary tract (men only), sarcoma, eye melanoma, mycosis fungoides) and the control groups, or for availability of the code. All requests for accessing data of the rare cancer case–control study are discussed on a case-by-case basis by the Steering Committee, e.g., to check whether the intended use is in agreement with the consent given by the study’s participants. For this, interested parties are asked to provide details and aims (e.g., for testing the reproducibility of results) on the purpose of their request. Any user must commit himself/herself to comply with the confidentiality rules of the rare cancer study, to not disclose any data to third parties, and to not use the data for any other purpose than the agreed purpose in writing. Data from the rare cancer study can thus be made available via the research service of Statistics Denmark and following the Danish Data Protection Regulation.

## Appendix A

**Questionnaire regarding X-ray and radiotherapy.**

**Question IX: X-ray and radiotherapy.**

We are interested in numbers of examinations and not separate pictures taken.

Routine examinations or screening examinations for e.g., tuberculosis or mamma cancer should be included but no dental examinations or non-medical examinations in for example shoe shops.

If the interviewee does not know the disease (or suspected disease) for which he was X-ray examined, then ask for the part of the body examined.

I would now like to ask you some questions about various types of diagnostic or therapeutic X-rays which you might have had in the past.

1. During your life have you had more than 20 X-ray examinations? (Yes/No/Don’t know)

For what reasons were you X-ray examined? (ICD-8 coding should be done by the local principal investigator).

2. For each reason/condition ask:

How many X-ray examinations did you in total have for this condition? And “When did you have the first X-ray examination for this condition”.

Disease/condition/part of the body? Number of examinations? Age at first examination?

3a. Have you ever had an X-ray examination with injection of radioactive substances? (Yes/No/Don’t know)

If yes, how many such examinations did you have? (Number of examinations) N.

If yes, at what age did you have the first such examination? (Age at first examination).

4a. Have you ever had radiotherapy treatment (X-rays, radium, radioactive isotopes, including injection of radio iodine for thyroid diseases? (Yes/No/Don’t know)

If yes: For which condition? ICD-8 code: xxx.x

How many times have you been treated with radiotherapy? Number of radiation treatment (N, Don’t know).

At what age did you have your first radiotherapy treatment? (Age in years, Don’t know)

At what age did you have your last radiotherapy treatment? (Age in years, Don’t know)

VII. Medical history

13b. If gall stones: was the diagnosis verified by ultrasound or X-ray examinations? (Yes/No/Don’t know), Age at onset/treatment? (Age in years).
